# Early Brain Sensitivity to Word Frequency and Lexicality During Reading Aloud and Implicit Reading

**DOI:** 10.3389/fpsyg.2019.00830

**Published:** 2019-04-11

**Authors:** Luís Faísca, Alexandra Reis, Susana Araújo

**Affiliations:** ^1^ Department of Psychology and Educational Sciences and Centre for Biomedical Research (CBMR), University of Algarve, Faro, Portugal; ^2^ Faculdade de Psicologia, Universidade de Lisboa, Lisbon, Portugal

**Keywords:** N1 print tuning, early top-down modulation, reading aloud, implicit reading, word frequency, lexicality effects

## Abstract

The present study investigated the influence of lexical word properties on the early stages of visual word processing (<250 ms) and how the dynamics of lexical access interact with task-driven top-down processes. We compared the brain’s electrical response (event-related potentials, ERPs) of 39 proficient adult readers for the effects of word frequency and word lexicality during an explicit reading task versus a visual immediate-repetition detection task where no linguistic intention is required. In general, we observed that left-lateralized processes linked to perceptual expertise for reading are task independent. Moreover, there was no hint of a word frequency effect in early ERPs, while there was a lexicality effect which was modulated by task demands: during implicit reading, we observed larger N1 negativity in the ERP to real words compared to pseudowords, but in contrast, this modulation by stimulus type was absent for the explicit reading aloud task (where words yielded the same activation as pseudowords). Thus, data indicate that the brain’s response to lexical properties of a word is open to influences from top-down processes according to the representations that are relevant for the task, and this occurs from the earliest stages of visual recognition (within ~200 ms). We conjectured that the loci of these early top-down influences identified for implicit reading are probably restricted to lower levels of processing (such as whole word orthography) rather than the process of lexical access itself.

## Introduction

People recognize written letters at such effortless and fast rate (<200 ms; Maurer and McCandliss, 2007), thanks to a universal, highly-specialized network specifically tuned to the recurrent properties of the orthographic code. This functional network comprises the left ventral occipitotemporal cortex and notably the visual word form area (VWFA; Cohen et al., 2002; McCandliss et al., 2003; Dehaene, 2010), whose responsivity to familiar letter strings (i.e., enhanced activation) originates from extensive experience with visual word forms. Event-related potential (ERP) studies have consistently identified the visual N1 (or N170) component as a neural correlate of fast, visual specialization for print (e.g., Bentin et al., 1999; Maurer et al., 2005b, 2006), presumably linked to the VWFA (Brem et al., 2006, 2009). The N1 follows the P1 component and is indicated by an enhanced negative deflection around 150–200 ms postpresentation of printed letters versus symbol strings or false fonts. A selective functional response of the N1 emerges rapidly with literacy acquisition (Maurer et al., 2006, 2007; Eberhard-Moscicka et al., 2015) and most impressively even after a short grapheme-phoneme training in kindergarten (Brem et al., 2010), neoliterate adults (Pegado et al., 2014), or adults trained on a novel script (Maurer et al., 2010). It is related to word-reading fluency (Eberhard-Moscicka et al., 2015) and reduced/missed in illiterates (illiterate vs. literate adults; Pegado et al., 2014) and poor readers (dyslexic vs. typically developing readers; e.g., Maurer et al., 2007; Araújo et al., 2012; Hasko et al., 2013). However, which cognitive processes exactly are involved and contribute to the early neural tuning for words, indexed by the N1, remains somehow unclear and is the purpose of the present study.

Developmentally, a “coarse neural tuning” for print establishes early in the course of learning to read (after only 1 year of reading instruction; e.g., Zhao et al., 2014; Eberhard-Moscicka et al., 2015), as indexed by the N1 difference in the ERPs between letter strings and visually similar nonletters (e.g., A vs. Ϫ). Though this N1 activation reflects a low-level specialization for visual aspects of print, it is linguistically modulated and, hence, tends to be left-lateralized in expert readers (Bentin et al., 1999; Pegado et al., 2014). This occurs because constant print-to-speech pairing during literacy acquisition establishes interconnections between left-hemisphere regions associated with phonological processing and occipito-temporal regions related to visual recognition of print (Maurer and McCandliss, 2007). The developmental trajectory for the enhanced sensitivity to visual words follows an inverted U-curve with initial increase and its subsequent decrease with age (Maurer et al., 2006; Brem et al., 2009). This change over time is probably due to acquired efficiency and full specialization involving more selective brain processes. For instance, a “fine-tuned” N1 for words emerges as reading acquisition progresses, strongly allied to reading ability, and N1 becomes then responsive to familiar orthographic patterns within words (e.g., BSNEO vs. BESNO for portuguese; Hauk et al., 2006; Zhao et al., 2014; Araújo et al., 2015).

An open question is whether the N1 just reflects an automatic, bottom-up response to surface form features (e.g., visual word form) or is it already sensitive to the activation of specific representations *within* the word recognition system. To address this, several studies have compared the brain’s neurophysiological response to two psycholinguistic dimensions of words known to influence lexical dynamics, word frequency, and word lexicality. Yet, results have been mixed: they either found larger N1 negativity in the ERP to low frequency words compared to high frequency words in adults (frequency effect; Sereno et al., 1998, 2003; Assadollahi and Pulvermuller, 2003; Hauk and Pulvermüller, 2004), reflecting the difficulty of accessing the lexical representations of low frequency words, or found no reliable effects in children (Araújo et al., 2012). Concerning lexical status, it has been shown that pseudowords elicited stronger brain responses than words in adults (lexicality effect; Hauk et al., 2009, 2012) and adolescents (Taroyan and Nicolson, 2009) already in an early time window. But again, lexicality effects on N1 have not been reliably found in children (Kast et al., 2010; Araújo et al., 2012; Hasko et al., 2013; Eberhard-Moscicka et al., 2015). All together, these results seem to suggest that N1 sensitivity to word frequency and lexicality depends on the phase of reading development, as well as on reading expertise (Araújo et al., 2015; Eberhard-Moscicka et al., 2015, 2016). However, in other studies, neither adults nor children processed pseudowords differently than words in the N1 component (Maurer et al., 2005b) or adults did not exhibit a N1 specialization for words over pseudowords in contrast to children who showed larger amplitudes for words (Maurer et al., 2006). It is possible that, beyond developmental aspects, factors such as reading strategies and task characteristics may contribute to or conversely mask differences in N1 sensitivity.

Previous studies have used different kinds of stimuli (real words of high- and low-frequency, pseudowords), but whether they trigger different reading strategies cannot be established based on these general stimulus categories. This is important given that the differences in reading strategies (from letter-by-letter decoding to fluent whole-word reading), observed during the process of learning to read (Yoncheva et al., 2010; Ben-Shachar et al., 2011) or at different levels of proficiency (Zhao et al., 2014), potentially shape the N1 specialization for words. For example, when learning a new script, using grapheme-to-phoneme conversion for reading induces a more left-lateralized negativity in the N1 window relative to whole-word recognition (Yoncheva et al., 2010)^1^. Ben-Shachar et al. (2011) also provided longitudinal evidence (7-to 15-year-old children) that changes in BOLD signals in the left occipito-temporal sulcus, in the vicinity of the VWFA, correlates with the change in sight word efficiency (number of frequent words read in 45 sec) but not with raw scores in phonemic decoding efficiency (pseudoword reading). But perhaps when reading becomes highly automated, like in proficient adult readers, print tuning disengages from reading strategies modulation (cf. Maurer et al., 2010). The present study followed up on this idea, aiming at testing adults’ N1 sensitivity to lexical word properties (word frequency and lexicality) within a paradigm where the design and the stimulus material were carefully selected to elicit the presumable use of different reading strategies, either by whole-word recognition vs. piece-wise grapheme-to-phoneme conversion (see below).

A related question is whether and how the linguistic intention of the subject (given the task goals) could affect N1 sensitivity to the lexico-semantic properties of a written word. To date, mainly implicit word-processing tasks were used to study early visual processing, such as repetition detection (e.g., Maurer et al., 2006; Eberhard-Moscicka et al., 2015, 2016), lexical decision (a general measure of “wordlikness”, e.g., Kast et al., 2010; Mahé et al., 2012), or other variants of implicit reading (Araújo et al., 2012, 2015). However, using these implicit tasks as a proxy of reading in real life may not be as straightforward: in these tasks, participants had no conscious intention to engage in linguistic processing, and the focus is presumably on visual word form rather than grapheme-to-phoneme conversion. Moreover, though implicit reading is usually effective in activating the reading network (e.g., Ben-Shachar et al., 2011), different electrophysiological patterns emerge just after the low-level visual analysis when processing print stimuli during implicit versus explicit reading tasks (with silent reading: Chen et al., 2013; with reading aloud: Mahé et al., 2015). This is (at least partly) expected given the demonstrations that even automatic/unconscious perception of stimuli can be modulated by context (e.g., stroop effect; Besner et al., 1997; masked priming N400 effects; Kiefer and Martens, 2010).

Therefore, in recent years, a few electrophysiological studies have explored the effects of task demands (e.g., could involve grapheme-phoneme decoding or simple visual recognition) on the processing of surface features (e.g., word form; Wang and Maurer, 2017; Sánchez-Vincitore et al., 2018) and of lexico-semantic properties of a word (Chen et al., 2015; Mahé et al., 2015; Strijkers et al., 2015) at the earliest latencies. For instance, Strijkers et al. (2015) observed an effect of word frequency as early as 120 ms after stimulus onset when readers consciously retrieved the meaning of the words (semantic categorization), but not until 100 ms later (at around 220 ms), when participants categorized the colored font of the same words (ink color categorization, where no linguistic processing is necessary). Recently, Wang and Maurer (2017) extended these findings by showing that task demands influence coarse neural tuning for print in the (late part of) N1, i.e., the letter-symbols difference was more pronounced in delayed naming and color detection compared to repetition detection. Taken together, these findings suggest that, though word recognition processes are largely automatic in the brain, very early on (N1 time window) visual-orthographic processing is flexible and penetrable to top-down influences. But very little attention has been dedicated to examining how these findings extend to the intentional and conscious skill of reading, a more ecological task.

Only a few studies have used explicit reading tasks and mainly to evaluate coarse neural tuning for print (Yoncheva et al., 2010; Chen et al., 2013; Sánchez-Vincitore et al., 2018; but see also Chen et al., 2015 and Mahé et al., 2015). For example, a recent study suggested a stronger sensitivity to word frequency in a lexical decision task compared to the silent reading task, reflected by enhanced activation of the ventral occipito-temporal cortex around 160 ms (Chen et al., 2015; but see Mahé et al., 2015). This result suggests that top-down modulation already affects information retrieval processes in visual word recognition and also in decision processes.

The present study thus aimed to further investigate (1) the influence of lexical word properties on the very early stages of visual processing (< 250 ms) of written words, and (2) whether the earliest modulation by lexico-semantic information retrieval (if any) interacts with task demands (i.e., the type of processing strategies required by the task, either grapheme-phoneme decoding for ulterior production or simple visual recognition for immediate-repetition detection). For (1), we manipulated the word form frequency (high vs. low) and the lexical status (real words vs. pseudowords) of the written words, all being well-matched for important sublexical aspects. Critically, we wanted to take this manipulation a step further, i.e., we ensured that words either encouraged alphabetic decoding versus whole word recognition for reading. Thus, stimuli were selected after being previously tested in an independent reading task with eye-movement recordings: supposedly, the reader’s spatial and temporal approach to the word provides a proxy of the reading strategies used (Hawelka et al., 2010; Schattka et al., 2010; see Method section). In addition, we used a blocked list design in order to exacerbate early differences tied to reading strategies. It is conceivable that the block-wise design favors lexical processing for words versus grapheme-phoneme conversion as the preferred unit of phonological recoding for pseudowords (Kinoshita et al., 2004; Pagliuca et al., 2007; Lima and Castro, 2010). For testing (2), we compared the brain’s response to print in the context of a task where conscious linguistic processing is not mandatory (one-back task as a measure of implicit reading) versus a more ecological task (delayed reading aloud task^2^) that required explicit reading and minimizes effects related to visual short-term memory or to task dependent decision/verification processes, testing the same participants and material in both tasks. We argue that the most convincing evidence in terms of specific word recognition processes will come from studies with complementary designs. This was the motivation and aim of our study. Typically developing adult readers have already reached automaticity in reading; therefore, we expect to observe a predominant left-lateralized N1 for all stimuli (words and pseudowords), irrespective of the task. Moreover, if lexical access during word recognition is instantiated automatically in adult readers, we predict lexical effects to start already around the N1 time window. Any interaction with task at these latencies would provide evidence for top-down task modulation of early retrieval of specific psycholinguistic information.

## Materials and Methods

### Participants

Thirty-nine adults (27 females) aged between 17 and 32 years (mean age [±SD] = 21.7 [±3.1] years) participated in this study. They were all undergraduate students and Portuguese native speakers and did not report neurological diseases or psychiatric disorders neither had history of reading and/or spelling problems (Portuguese adaptation of the Adult Reading History Questionnaire; Alves and Castro, 2004). Additional inclusion criterion for all the participants was a nonverbal IQ in the normal range (>85; Wechsler Adult Intelligence Scale—WAIS-III) and adequate reading level as determined by a reading decoding and comprehension test for dyslexia screening (Lobrot L3 > 25th percentile; 1-min time limit; five alternative forced-choice of the word that completes a sentence; total of 36 sentences; Portuguese adaptation for adults: Fernandes et al., 2017). Moreover, a reading aloud fluency test of the Differential Diagnosis Dyslexia Battery (3-DM, Portuguese version: Pacheco et al., 2014) was applied. This test comprised three lists of high-frequency words, low-frequency words, and pseudowords. Performance is computed as the number of stimuli read correctly per list in 30 s (mean score for real-word reading composite, *M* = 2.0 items/sec, *SD* = 0.28; for pseudoword reading, *M* = 1.5 items/sec, *SD* = 0.22). Data from ten additional subjects were excluded either due to poor reading level (three participants) or excessive movement and eye blinking artifacts or other technical problems during EEG recording (seven participants). All participants gave their written informed consent to participate in the study and were paid for compensation.

### Stimuli Material

The same material was used both for the one-back task and the reading aloud task. A total of 100 words (50 high-frequency words—HFW and 50 low-frequency words—LFW) were selected according to their word-form frequency (frequency of occurrence per million, *M* = 125.1 vs. 0.7 for high-frequency vs. low-frequency; P-PAL database; Soares et al., 2018). Fifty orthographically legal and pronounceable pseudowords (PW) were also created by exchanging at least two letters in the set of real words. Words and pseudowords were four-to-nine letters long, and all three conditions were matched (*F* tests, all *ps* > 0.2) in orthographic and phonological length, bigram frequency, and orthographic neighborhood density.

Important, the current study for the first time controlled for the reading strategies elicited by different words by means of eye movement recording. That is, all stimuli (high- and low-frequency words and pseudowords) to be included were selected after being previously tested in an independent reading task with 40 undergraduate students, while eye movements were recorded (SMI hi-speed eye tracking system, 1,250 Hz; see Silva et al., 2016, for a detailed description of the paradigm). In this task, words were arranged in six sets of matrices corresponding to the orthogonal manipulation of familiarity (high- and low-frequency words and pseudowords) and word length (short, long); each matrix comprised 12-to-15 items arranged in a 3 × 4/5 layout and 5 matrices for each set were presented (in total, 80 × 3 experimental stimuli plus fillers). Participants were instructed to read these words in a left-to-right and down fashion, and their speech responses and eye-movements were collected. Eye-movement data provide a good indication of online cognitive processing during reading such as the ease or difficulty of visual word recognition (Rayner, 1998) and might be informative about the reader’s processing strategy, either a sublexical strategy or a lexical strategy for reading. For example, the well-documented word length effect in the case of unfamiliar words is an important marker of sublexical strategies manifested in RTs and, notably, also on the eye tracking parameters (that is, longer gaze duration and higher number of fixations for long items compared with short items; e.g., Hawelka et al., 2010). Thus, the assumption here was that prolonged gaze durations and higher fixation counts for words are taken to reflect sublexical decoding-based processes. In contrast, single fixations and shorter gaze durations, expected for the easiest items (i.e., familiar visual words), are suggestive of lexical reading *via* direct orthographic whole-word recognition (Hawelka et al., 2010; Schattka et al., 2010; Ablinger et al., 2014). For the present study, the selected PW received a significantly higher number of fixations and longer gaze durations (*M* ± *SD* = 3.26 ± 1.02 and 877 ms ± 253) than the selected LFW (*M* ± *SD* = 2.48 ± 0.52 and 610 ms ± 130) and those with HFW (*M* ± *SD* = 1.79 ± 0.31 and 422 ms ± 66), with stimulus length controlled; all differences between conditions were highly significant (*p* < 0.001). We thus assumed that participants rely on different reading strategies when processing these different types of words. Moreover, given that stimulus conditions were presented in separate blocks (see below), it is likely that the words-only presentation biases toward lexical processing, while the pseudowords-only list elicits a stronger reliance on smaller units of phonological recoding (e.g., Pagliuca et al., 2007; Lima and Castro, 2010).

### Experimental Procedures

**Figure 1:**
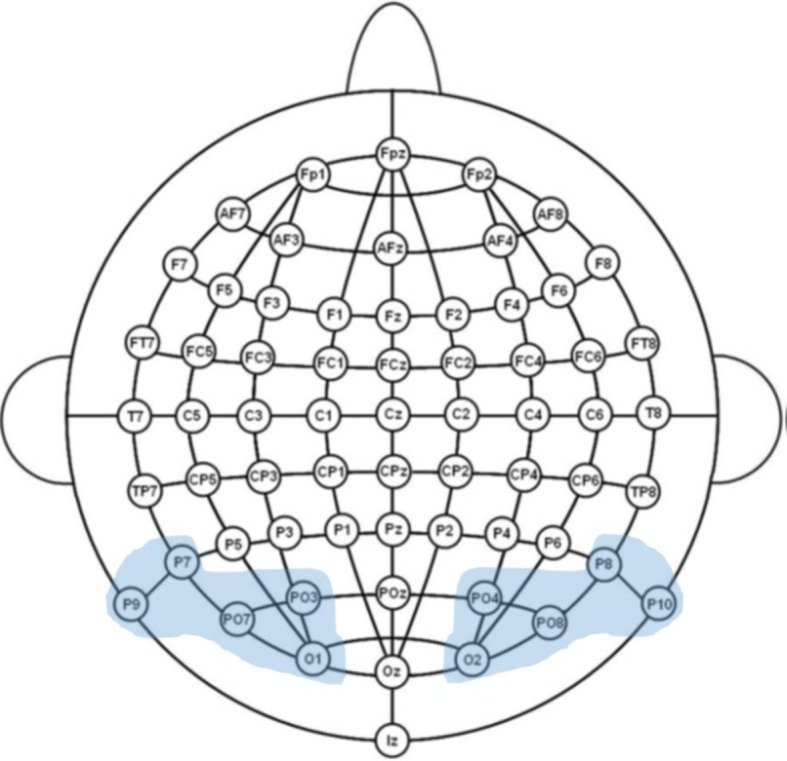
Illustration of the 64-channel system used in the experiment and the examined regions of interest.

Each task was split into three blocks of HFW, LFW, and PW presented in pseudorandom order with specific instructions and a brief training (eight practice trials) before each block (Figure 1). The sequence of blocks was counterbalanced between participants. For the *explicit reading task,* we used a delayed reading aloud format to prevent recordings from being contaminated by speech-related artifacts. Hence, this task allowed ERPs to be calculated for each stimulus on its initial presentation without interference from any reaction on the part of the subject, while behavioral accuracy responses after stimulus presentation ensured that subjects were engaged in the task. Each trial began with a fixation cross (500 ms) which was then replaced by a blank screen (100 ms), followed by the stimulus for 800 ms. Then, participants were cued with question marks “???” (1,500 ms) to read aloud the preceding (pseudo)word. The next trial began after an intertrial interval of 1,500 ms (including a period for the participants to blink their eyes). Participants were asked to pay attention to the words and pseudowords displayed but only to read them out loud whenever they saw question marks.

For the *implicit reading task*, we used a one-back task that has been commonly used in EEG research on early visual word recognition. Participants were asked to watch sequences of words and pseudowords and to press a button whenever an immediate repetition occurred (17% of the time); they were not required to read consciously the stimulus being presented. Each trial was presented on the following sequence: firstly, a fixation cross (500 ms) was displayed, which was then replaced by a blank screen (100 ms). Then, the stimulus appeared for 800 ms. Again, the next trial began after an inter-trial interval of 1,500 ms. In both tasks, all (pseudo) words were displayed in lower case, in black Arial font on a white background, at eye-level at the center of the screen, and ranged from 2.2° to 3.8° visual angle.

The participants were tested individually in a soundproof room and sat at ~100 cm in front of a computer screen, being instructed to remain still and relaxed. Presentation software (version 11; https://www.neurobs.com/) was used to display the stimuli and record the participant’s responses for the one-back task. The spoken responses in the reading aloud task were digitally recorded for latter response accuracy check.

All participants completed both tasks^3^ in counterbalanced order. Previous analyses conducted with task order as a factor yielded no main effects or interactions, and so task order was collapsed for the reported analyses.

### EEG Recording and Analysis

**Figure 2:**
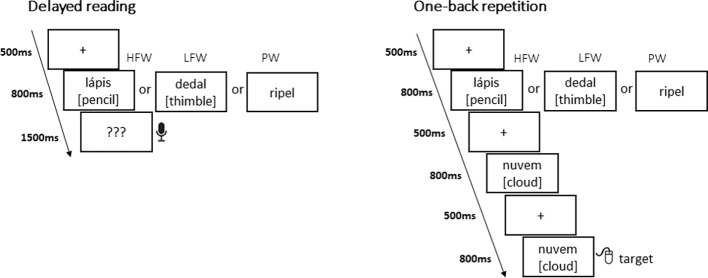
Examples of stimuli and presentation sequences in the delayed reading task and in the one-back repetition task (50 items were presented per experimental condition in a blocked design with high-frequency words—HFW, low-frequency words—LFW, and Pseudowords—PW).

The electroencephalogram (EEG) was recorded continuously using an ActiveTwo Biosemi amplifier (DC-67 Hz bandpass, 3 dB/octave, 24-bit sampling, 512 Hz sampling rate) from 64 Ag/AgCl scalp electrodes mounted in an elastic cap according to the International 10–20 system guidelines. The electrode montage included 10 midline sites and 27 sites over each hemisphere (Figure 2). Additional electrodes were used as ground and online reference (CMS/DRL nearby Pz; for a complete description, see biosemi.com) and for recording the electroencephalogram (EOG; placed below the right eye).

The EEG data were analyzed using the FieldTrip open source toolbox (Oostenveld et al., 2011). The continuously recorded data were epoched from −125 before to 700 ms following presentation of the stimulus and were time-locked to the onset of the target stimuli. Offline, the EEG data were low-pass filtered at 30 Hz and transformed to an average reference (eye electrodes were excluded to compute the common reference), and a baseline correction was applied by subtracting the average pre-stimulus voltage from the entire waveform. Bipolar EOG was computed using the Fp2 and the electrode placed vertically (vertical eye-movements) and horizontally using the F7 and the F8 electrode. Before averaging, epochs for each participant were physically inspected and those containing blinks and horizontal eye movements, muscle, or other artifacts were manually removed from the analysis. Data were visually artifact rejected on a trial-by-trial basis for eye blink and on a channel-by-channel basis for drift, blocking, and excessive alpha wave; the rejection procedure was blind to participants and conditions. A minimum of 30 trials for each of the conditions, per participant, were included in the final analyses. ERP data were analyzed by computing the mean amplitude of the waveforms during specific time windows, relative to the −125 to 0 ms pre-stimulus baseline interval.

All corrected trials were first averaged within experimental condition for each channel, synchronous to the onset of the target and following baseline correction. To restrict the number of statistical comparisons, a region-of-interest (ROI) approach (i.e., data averaged over a sub-set of electrodes, selected *a priori* according to theoretical considerations and visual inspection) was then used to calculate a grand-average over all participants for each condition and time window of interest.

To investigate fine-tuning effects in early visual processing, we analyzed brain’s sensitivity to word form frequency and lexicality during the time windows from 90 to 120 ms (P1 component), given that prior studies have identified this component as the earliest index of specialized orthographic processing (e.g., Maurer et al., 2005a, 2006) and from 160 to 220 ms (N1 component). The mean amplitude of the Word frequency effect (high-frequency *vs*. low-frequency) and the Lexicality effect (low/high-frequency *vs*. pseudowords) on a set of representative sites (P7/P8, P9/P10, PO7/PO8, PO3/PO4, O1/O2) was subjected to an omnibus repeated measures ANOVA, including the factors Task (implicit reading vs. explicit reading), Stimulus type (HFW vs. PW and LFW vs. PW), and Hemisphere (right parieto-occipital sites vs. left parieto-occipital sites). Whenever two- and three-way interactions involving Task were found to be significant, we proceed to test each contrast regarding our manipulation of interest separately in a mixed-design ANOVA.

As a complementary approach, we performed a systematic analysis of our main component of interest (early N1 ERP component) in peak time window by using mean amplitude over +/− 30 ms interval around the maximum peak (determined per subject for each condition and for the clusters of channels of interest).

## Results

### Behavioral Results

To assess differences in difficulty between the explicit and implicit reading tasks, we ran repeated measures ANOVA on the error percentages with Task (implicit reading and explicit reading) and Stimulus (HFW, LFW, and PW) as within-subject factors. Accuracy was close to ceiling for both tasks, although slightly higher for the explicit reading task (implicit reading task: *M ± SD* = 93.4% ± 7.8; explicit reading task: *M* ± *SD* = 97.0% ± 2.1%; *F*(1, 38) = 7.2, *p* = 0.011, *partial-ƞ^2^* = 0.16). Given these high accuracy responses, further differences in evoked brain responses between both tasks are not likely related to poor accuracy in performing the task or task comprehension difficulties. A significant interaction suggests that accuracy differences between stimulus were not equal for both tasks, *F*(2, 76) = 7.6, *p* < 0.001, *partial-ƞ^2^* = 0.17: while error rates were similar for the three type of stimulus in the implicit reading task (*p* = 0.935), for the explicit reading task, HFWs were more often correctly named (*M* = 99.9%) than LFW (*M* = 97.6%) and both more correctly named than PW (*M* = 93.5%), *F*(1.4, 51.4) = 62.5, *p* < 0.001, *partial-ƞ^2^* = 0.62, with Greenhouse–Geisser correction for sphericity.

### Electrophysiological Results

#### Sensitivity to Word Form Frequency

To test the P1–N1 sensitivity to word-form frequency, we contrasted ERPs to letter-strings that mainly differ by frequency of occurrence. An overall analysis was done with Task (implicit reading vs. explicit reading), Stimulus Type (HFW vs. LFW), and Hemisphere (right parieto-occipital sites vs. left parieto-occipital sites) as within-subject factors.

*P1 (90–120ms):* Only a main effect of Hemisphere was observed at around 90–120 ms, *F*(1,38) = 13.3, *p* < 0.001, *partial-ƞ^2^* = 0.26, revealing that at posterior sites, the P1 elicited by high- and low-frequency words was more positive over the right than the left hemisphere. We did not find reliable Stimulus (*p* = 0.215) and Task (*p* = 0.908) effects (all interactions involving these factors, *p’s* > 0.4).
*N1 (160–220ms):* In the 2 (Task) × 2 (Word form frequency) × 2 (Hemisphere) omnibus ANOVA run on the N1 mean amplitude, the three-way interaction Stimulus by Task by Hemisphere was at a trend level, *F*(1, 38) = 3.9, *p* = 0.055, *partial-ƞ^2^* = 0.09. Planned comparisons were then performed for each task separately. The main effect of hemisphere was robust for both implicit reading, *F*(1, 38) = 8.6, *p* = 0.006, *partial-ƞ^2^* = 0.18, and explicit reading, *F*(1, 38) = 9.5, *p* = 0.004, *partial-ƞ^2^* = 0.20. As expected, ERPs were more negative over the left parieto-occipito sites than the right parieto-occipito sites. The effect of Word frequency did not reach statistical significance (explicit reading: *F*(1, 38) = 2.7, *p* = 0.107, *partial-ƞ^2^* = 0.07; implicit reading: *p =* 0.676), hence indicating no significant difference in processing high- and low-frequency words irrespective of the task. Neither did the interaction of Word frequency and Hemisphere (for both tasks, *p’s* > 0.2).

The same analysis was repeated using the window centered at the N1 peak. Again, only the main effect of hemisphere was significant, *F*(1, 38) = 10.1, *p* = 0.003, *partial-ƞ^2^* = 0.21. The main effect of Word frequency and the interaction Word frequency by Task were still nonsignificant (all *p’s* > 0.4).

**Figure 3:**
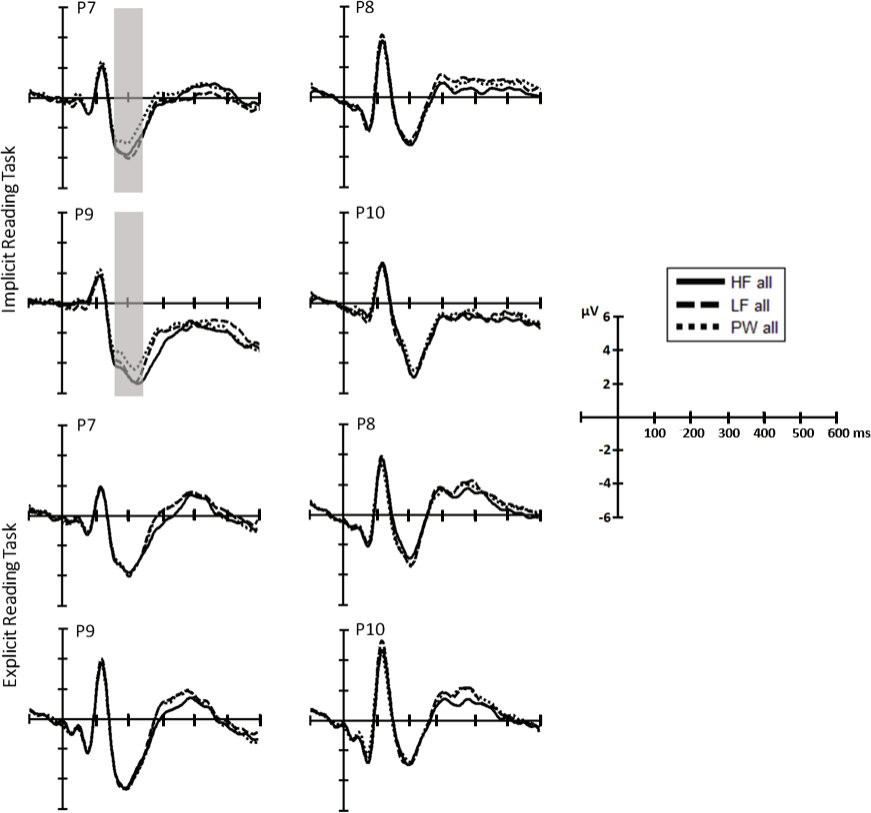
Separate ERP waveform comparison in each task (top, repetition detection; bottom, reading aloud). ERP waveforms at representative electrodes for high-frequency (solid line) and low-frequency words (dashed line) and pseudowords (dotted line).

Yet, visual inspection of Figure 3 suggested the possibility of an effect of word frequency on later stages of processing at around 300 ms that already start during the N1. Indeed, when we analyzed voltages on this later time window, just after the N1 (220–340ms after stimulus onset), word frequency did affect brain responses, *F*(1, 38) = 6.9, *p* = 0.012, *partial-ƞ^2^* = 0.15, as high-frequency words yielded larger amplitudes than low-frequency words (main effect of hemisphere, F(1, 38) = 4.7, *p* = 0.036, *partial-ƞ^2^* = 0.11, indicating larger negativity at the left posterior sites). No main effects of Task or interactions of interest were observed in this later time window (all *p’s* > 0.3).

#### Sensitivity to Lexicality

To investigate early effects of whole-word processing (sensitivity to lexicality), we contrasted the brain activation to real words and pseudowords. We run two separate ANOVAs: one contrasting HFW vs. PW and the other one LFW vs. PW. For both analysis, the factors were Task (implicit reading vs. explicit reading), Stimulus type (HFW vs. PW or LFW vs. PW) and Hemisphere (right parieto-occipital sites vs. left parieto-occipital sites).

**Figure 4:**
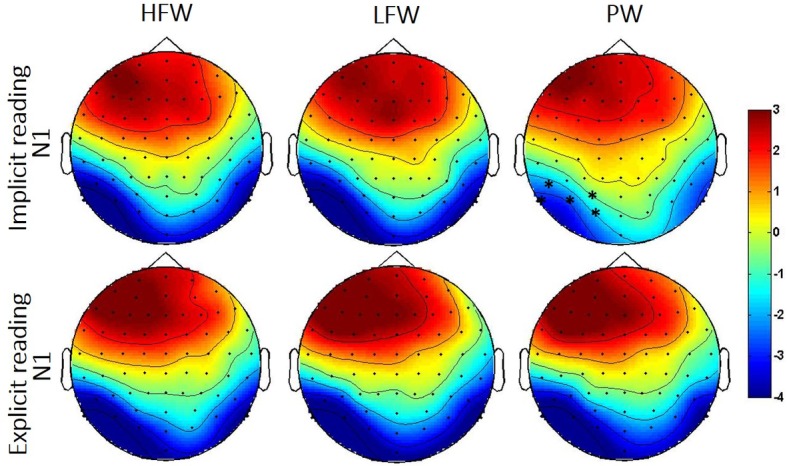
Topographic distribution of the N1 effects in the implicit and explicit reading tasks per experimental condition (HFW, high-frequency words; LFW, low-frequency words; PW, pseudowords). The shaded rectangles indicate the distribution of recording electrodes (Biosemi Active-Two system with 64 channels). Significant cluster for the low frequency/high-frequency words vs. pseudowords contrasts is highlighted by black asterisk (*).

*P1 (90–120ms*): The two-way interaction between Stimulus type and Hemisphere was significant in both ANOVAs (HFW vs. PW: *F*(1, 38) = 5.7, *p* = 0.022, *partial-ƞ^2^* = 0.13; LFW vs. PW: *F*(1, 38) = 6.0, *p* = 0.019, *partial-ƞ^2^* = 0.14). Post-hoc pairwise comparisons showed that at the right hemisphere ERPs elicited by high- and low-frequency words were more positive than those elicited by pseudowords (*p* = 0.015 and *p* < 0.001, respectively) while there was no lexicality effect for the left hemisphere. The effect of Stimulus was independent of the Task (Stimulus by Task: HFW vs. PW—*F*(1, 38) = 1.5, *p* = 0.224, *partial-ƞ^2^* = 0.04; LFW vs. PW—*F*(1, 38) = 2.7, *p* = 0.107, *partial-ƞ^2^* = 0.07; three-way interactions, both *p’s* > 0.3).
*N1 (160–220ms):* The omnibus ANOVAs revealed that task demands interacted with the stimulus effect in the N1 time window as shown by the three-way interaction Stimulus, Task and Hemisphere (LFW vs. PW: *F*(1, 38) = 5.6, *p* = 0.023, *partial-ƞ^2^* = 0.13) and by the nearly significant interaction between Stimulus and Task (HFW vs. PW: *F*(1, 38) = 3.1, *p* = 0.088, *partial-ƞ^2^* = 0.08) (see Figures 3 and 4). Planned comparisons separately by Task indicated a Lexicality effect on the left hemisphere for the implicit reading (HFW vs. PW: *F*(1, 38) = 5.8, *p* = 0.021, *partial-ƞ^2^* = 0.13; LFW vs. PW: *F*(1, 38) = 11.4, *p* = 0.002, *partial-ƞ^2^* = 0.23). HFW and LFW elicited more negative-going ERPs compared to PW over the left occipito-parietal sites, while at right sites, the N1 mean amplitudes did not differentiate processing between stimulus. However, for the explicit reading task, we found no difference between real words and PW (HFW vs. PW: main effect of Stimulus, *p* = 0.736, and Stimulus by Hemisphere, *F*(1, 38) = 2.3, *p* = 0.140, *partial-ƞ^2^* = 0.06; LFW vs. PW: main effect of Stimulus, *F*(1, 38) = 1.1, *p* = 0.296, *partial-ƞ^2^* = 0.03, and Stimulus by Hemisphere*, p* = 0.998). For this task, only the main effect of Hemisphere reached significance in both ANOVAs (HFW vs. PW: *F*(1, 38) = 11.3, *p* = 0.002, *partial-ƞ^2^* = 0.23; LFW vs. PW: *F*(1, 38) = 8.4, *p* = 0.006, *partial-ƞ^2^* = 0.18), with ERPs being more negative over the left than the right hemisphere.

Additionally, we performed the same repeated measures ANOVAs on the peak of the N1. We found that lexicality effects are modulated by task, i.e., implicit reading was associated with greater left posterior activation for real words versus pseudowords (HFW vs. PW: *F*(1, 38) = 9.1, *p* = 0.005, *partial-ƞ^2^* = 0.19, LFW vs. PW: *F*(1, 38) = 14.5, *p* < 0.001, *partial-ƞ^2^* = 0.28), while lexicality effects were observed for the explicit reading (both ANOVAs, *p’s* > 0.3).

## Discussion

This study aimed to explore whether lexical information of a word (i.e., word frequency and lexical status) influences the early stages of visual word recognition and if this influence depends upon the task demands. We recorded ERPs during two reading tasks that either necessarily involve linguistic processing (delayed reading aloud) or not (one-back repetition detection) and using strictly the same material (high-frequency *vs.* low-frequency words *vs.* pseudowords) and participants in both tasks. In this study, we refrained from testing coarse neural tuning for print, as indexed by differences in amplitudes between letter and symbols strings (therefore, symbols were not included in the material). Robust print tuning effects in the visual N1 have already been demonstrated elsewhere, at the group (e.g., Maurer et al., 2006, 2007; Brem et al., 2009; Araújo et al., 2012) and individual level (Eberhard-Moscicka et al., 2016). However, studies do not agree in finding differences between different kinds of letter strings such as lexicality and frequency effects. These effects were then the focus of the present study and our core findings were (1) a robust left-lateralized N1 response in adult expert readers that generalizes to different letter string categories and tasks, (2) early lexicality effects that are task-dependent, and (3) absence of word frequency effect at the early P1-N1 time windows, irrespective of the task (a late frequency effect was rather found, around ~300 ms).

### Lateralization of N1

In the N1 component, we found larger negativities at left compared to right posterior sites across all types of letter-strings and irrespective of the task. This left-lateralization of N1 for word stimuli is expected for fast, automatic linguistic processes in skilled readers, as opposed to right-hemispheric topography of the N1 in children and adults with low literacy skills, presumably more linked to visual familiarity effects (Maurer et al., 2005b; Sánchez-Vincitore et al., 2018). Interestingly, these task-independent lateralization effects in skilled adult readers contrast with prior studies of adults learning a novel script: “words” trained through grapheme-to-phoneme conversion elicited left-lateralized N1 responses to the reading verification task (Yoncheva et al., 2010) but not to the one-back task (Maurer et al., 2010). Hence, the type of processing strategies required by the task (i.e., task demands) influences lateralized processes linked to perceptual expertise for reading within ~200 ms but apparently on an earlier acquisition stage. That is, explicit attention on orthography-to-phonology associations may be a necessary condition for a left-lateralized N1 response to visual words in early phases (cf. Maurer et al., 2010). As readers become more expert-like, a predominantly left-lateralized engagement is elicited, not modulated by attention and task demands as observed here (see also Strijkers et al., 2011b).

### Sensitivity to Lexicality and Word-Form Frequency

In what regard lexical dynamics, the effects of lexicality and word frequency in early stages of visual word recognition have been volatile: from significant effects in adults but not in children (Hauk et al., 2009, 2012; Eberhard-Moscicka et al., 2016) or the reverse (Maurer et al., 2006) to null effects (Maurer et al., 2005b). Moreover, these effects have been barely investigated with explicit reading tasks. Here, we replicated the finding that in adult readers, lexical processing already happens within the first 220 ms of viewing the words during implicit reading (e.g., Hauk et al., 2012; Araújo et al., 2015; Eberhard-Moscicka et al., 2016): using a one-back task, we observed that N1 was increased for real words compared to pseudowords, probably reflecting greater sensitivity for familiar orthographic patterns. Alternatively, though the one-back task minimizes deliberate higher-order processes, task-unrelated automatic phonological activation of words may still have occurred for a certain extent (Kronschnabel et al., 2013). These early lexicality effects have not been seen for younger children (Eberhard-Moscicka et al., 2015, 2016). Yet, we note that a functional relation between the “lexical” N1 specialization in adult readers and (proficient) reading skills was not observed in our data: the word-pseudoword N1 effects at the left hemisphere did not correlate with word reading fluency (correlation with HFW-PW difference: *r* = 0.04, *p* = 0.825; correlation with LFW-PW difference: *r* = −.23, *p* = 0.157). This null finding might suggest that the N1 word form-sensitivity in competent readers reflects a process which is already highly automatized. Accordingly, prior ERP data had shown that the N1 specialization follows a nonlinear development (e.g., Brem et al., 2006).

On the other hand, when conscious linguistic processing is mandatory, as in explicit reading, the activation elicited by words and pseudowords was similar at the N1 time window. This null effect for lexicality replicates earlier findings (Mahé et al., 2015). Hence, we found evidence supporting a task effect on the early neural processes involved in reading: a psycholinguistic variable such as lexicality exerted an influence on early visual word processing but the pattern of its influence was sensitive to the task demands placed on the reader. That is, when the task did not require explicit reading (as for visual immediate-repetition detection), the ERPs elicited by words displayed more negative-going amplitudes at the left hemisphere when compared to those elicited by pseudowords; this suggests that when the task requires a shallow processing (simple visual recognition), real words might engage automatic reading-related processes to a larger degree than pseudowords do (Maurer et al., 2005a; Eberhard-Moscicka et al., 2016), possibly due to their extensive exposure and a tight relationship with phonology. However, these automatic reading processes seem to be flexible enough to accommodate the task demands such as when explicit reading is required. The absence of lexicality effects in our delayed reading aloud task suggests that the goal of the task (reading aloud both words and pseudowords) modulates reading processes, focusing participants’ attention to the grapheme-phoneme decoding attributes of the stimulus.

Though less robust, some of the differences found in the N1 already started earlier during the P1 time window (~100 ms), in agreement with some previous studies (Zhao et al., 2014). The P1 component has been associated with low-level visual processing but is also sensitive to attention load (e.g., Araújo et al., 2015), independent of the literacy level (Pegado et al., 2014). Thus, the lexicality effect observed at this first peak likely arises from a greater perceptual resource allocation for pseudowords than for words (as the visual processing demands are greater for the former), while neural signatures actually corresponding to lexical access occur slightly later, at the N1 window. Importantly, this lexicality effect observed in P1 time window was not modulated by the task. The absence of early task modulation at the level of P1 thus suggests that the interaction effects at N1 window cannot be explained by an exogenous increase in attention toward word stimuli specifically in the one-back repetition detection. Rather, it seems that different intentional goals for explicit reading versus immediate-repetition detection in this study may have induced strategic top-down modulations in processing of words versus pseudowords at early latencies of visual recognition. This early modulation either occurs through facilitating access to word representations, or, alternatively, the loci of these effects are restricted to lower levels of processing such as whole word orthography (see e.g., Katz et al., 2005, Experiment 3). In the former case, yet, we should have seen an earlier lexicality effect for explicit reading compared to when no reading intention is present, because the requirement to speak aloud instigates faster access to the lexicon (Strijkers et al., 2011a), which in turn should be harder for pseudowords.

In a related study, a task-driven lexicality effect was not found: Mahé et al. (2015) reported that from about 140 ms after perceiving a word, the adults’ brain electrical response dissociates between reading aloud and lexical decision (taken as a measure of implicit reading), which however did not depend on lexicality (a very late lexicality effect was found in both tasks). Thus, one factor that seems to be of importance is the depth of linguistic processing required in the implicit reading tasks, which may be stronger in lexical decision than in visual immediate-repetition detection of letter strings. By using the latter task, we and others (e.g., Eberhard-Moscicka et al., 2016) did observe an early lexicality effect. Alternatively, it is still possible that differences in the designs between Mahé et al. (2015) and our study may explain this discrepancy. Specifically, ERPs derived from block-wise presentations might be more affected by changes in the attentional states between words and pseudowords compared to randomized stimulus presentations (used in Mahé et al.’s study). However, we have no reason to suspect that attentional effects were more strongly enhanced for blocked words than for blocked pseudowords depending on the specific task.

It is possible that, beyond task demand and its interaction with lexical dynamics, the extent to which the reading strategies (lexical and sublexical) are engaged could per se modulate the N1 specialization (see e.g., Maurer et al., 2010; Ben-Shachar et al., 2011; Zhao et al., 2014). Experimental manipulations involving familiar words (emphasizing whole-word, lexical processing) and pseudowords (requiring letter-by-letter decoding; as predicted by dual-route models; e.g., Coltheart et al., 2001) are commonly used in reading research. But yet, in prior studies, we cannot rule out the possibility that due to shallow task demands (e.g., visual recognition in n-back), the participants processed these stimuli likewise, without recruiting different reading subprocesses (e.g., processing pseudowords as actual words, basing their decisions on “wordlikness”). The originality of the present study stands on the methodological control it offered, ensuring that the processing of the word and pseudoword stimuli is qualitatively distinct as based on external markers collected in an independent eye-tracking study (see method) and a blocked lists design (see e.g., Lima and Castro, 2010). Overall, our data add that, in the adult expert state, early print tuning disengages from reading strategies modulation, and therefore, the effect of stimulus type was null in explicit reading (where one would expect the effects of the reading strategies to be especially exacerbated). However, we support the notion that initial access to the linguistic system is influenced by task-driven top-down processes according to the behavioral goals that are relevant to specific tasks (Balota and Yap, 2006), either the intention to overt speech or not. This main outcome is at odds with the traditional view according to which any influence comes into play during late (post-)decision processes (e.g., Nobre et al., 1998; Bentin et al., 1999), while the observed effects can be accounted for in a number of ways within visual word recognition models (but which our study cannot truly disentangle). In principle, the evidence favors the assertion that some degree of feedback occurs in the system during visual word recognition, modulating early ERP markers. In an “interactive account” of reading, higher-level top-down (e.g., phonological) and visual bottom-up orthographic information interacts reciprocally and in an automatic fashion for visual word recognition (Price and Devlin, 2011). Accordingly, prior studies have provided evidence supporting early-top down effects from the lexical to the abstract orthographic/letter level of encoding (e.g., case match effects at around 200 ms interacted with lexicality in an identity priming paradigm: Vergara-Martínez et al., 2015). Or, our results could be predicted from the Bayesian modeling framework (Norris, 2006; Norris and Kinoshita, 2012), by assuming that readers behave as “optimal” decision makers that take into account perceptual evidence framed by prior knowledge (lexicality effects as an index of the higher probability of real words) combined with their goal and the decision to be made. This view does not necessarily imply feedback mechanisms during visual word recognition, but eventually task demands can tune some parameters of the visual word recognition system and, especially in a block design, shape the feedforward stream of information without requiring a continuous adjustment through feedback control (Norris et al., 2000). In a study of masked priming, for example, Norris and colleagues have shown an equivalent priming effect in *same* responses to nonwords during a same-different task as in *yes* decisions to words in lexical decision, expressed in behavioral and ERP data (Norris et al., 2018). This result was thus interpreted as indicating that priming effects were more so a consequence of the cognitive and perceptual decision/computation that participants must perform on the stimulus than of automatic processing (specifically lexical or semantic) elicited by reading a word. A few other recent ERP studies have also revealed that different intentional goals influence the processing of surface properties (Wang and Maurer, 2017; Sánchez-Vincitore et al., 2018) and also fine tuning for print (Chen et al., 2015; Strijkers et al., 2015), implying that a flexible lexical processing system may depend to some extent on the specific demands of the task. We extended these results to the intentional and conscious skill of reading aloud.

In this study, we could not find reliable N1 differences between high- and low-frequency words, as reported occasionally for adults (e.g., Araújo et al., 2015; Eberhard-Moscicka et al., 2016). A word frequency effect was only seen at a later stage of processing (~300 ms). Neither did we replicate the finding that linguistic intention leads to an earlier onset of word frequency effects (Strijkers et al., 2011a, 2015; Chen et al., 2015). A tentative explanation is that neural tuning for lexical familiarity improves over an inverted U-curve like the typical N1 coarse print tuning development (Maurer et al., 2006; Brem et al., 2009), and perhaps, ERP frequency effects are only observed upon certain conditions, e.g., depending on stimulus repetition or the list composition (specifically, “pure” lists of restricted frequency ranges vs. “mixed” word conditions modulate the word-frequency effect: Glanzer and Ehrenreich, 1979). It is also possible that the use of long words in our study (mean length: 6.8 letters) may have led to a slightly delayed onset of a stimulus frequency effect, given that the amplitude and specific latency of this effect at early brain responses (including the N1) might critically depend on word length. For example, using MEG, Assadollahi and Pulvermüller (Assadollahi and Pulvermuller, 2003) found effects of word frequency as early as 120-170 ms for short, monosyllabic words only (low frequency items leading to stronger brain responses) and latter frequency effects seen specifically for long words (5–7 letters), at around 240 ms.

To summarize, our results indicate that already within the earliest stages of processing, visual word recognition is open to influence from top-down processes due to the intention to engage in linguistic processing (reading aloud) or not (n-back repetition detection). These task-driven modulations extend beyond general word activation, as seen previously using “coarse” contrasts (all-word vs. resting period: Chen et al., 2013; words vs. symbols: Wang and Maurer, 2017), affecting also specific higher-order aspects of the word recognition process. In expert processing, this influence is (apparently) not modulated by reading strategies and is reflected by effects of lexicality within N1 in our study and extends to other psycholinguistic properties that affect lexical access (e.g., lexical frequency, imageability; Chen et al., 2015; Strijkers et al., 2015) tested using other task designs (more or less close to natural reading). However, lateralized reading processes associated with visual expertise for print-produced task-independent effects.

## Ethics Statement

The study followed the Portuguese Regulation for the Code of Ethics and Conduct in Psychology. All subjects gave written informed consent in accordance with the Declaration of Helsinki.

## Author Contributions

LF, AR, and SA contributed to the conception and design of the study. LF and SA organized the database and performed the statistical analysis. SA wrote the first draft of the manuscript. LF and AR wrote sections of the manuscript. All authors contributed to manuscript revision, read and approved the submitted version.

### Conflict of Interest Statement

The authors declare that the research was conducted in the absence of any commercial or financial relationships that could be construed as a potential conflict of interest.

## Footnotes

^1^It is worth noting that Yoncheva et al. (2010) refrained from relating their findings to a debate regarding dual reading routes (Coltheart et al., 2001), while the focus was on the importance of explicitly directing readers’ attention to small sublexical phonological units versus large (whole-word) units of representations on early reading acquisition.

^2^As we become competent readers, silent reading likely becomes a preferred reading mode. Furthermore, silent and oral reading not necessarily rely on the same underlying processes and strategies (e.g., Krieber et al., 2017). That said, reading aloud tasks (used in our study) can certainly still provide a good index of the processes occurring during “reading,” while they minimize effects related to visual short-term memory or to task dependent decision or verification processes.

^3^We acknowledge that our experimental design resulted in between task differences, with participants being requested to give a response to each trial in delayed reading and only to a limited number of trials in one-back detection. Nonetheless, in both tasks, ERPs were collected to each stimulus, i.e., during a period where participants prepared to responding in any case (and therefore in advance of the repetition/no repetition decision versus overt naming).
